# Problems of video-based pain detection in patients with dementia: a road map to an interdisciplinary solution

**DOI:** 10.1186/s12877-017-0427-2

**Published:** 2017-01-26

**Authors:** Miriam Kunz, Dominik Seuss, Teena Hassan, Jens U. Garbas, Michael Siebers, Ute Schmid, Michael Schöberl, Stefan Lautenbacher

**Affiliations:** 1 Physiological Psychology, Otto-Friedrich University Bamberg, Bamberg, Germany; 2 Department of General Practice, Section Gerontology, University of Groningen, University Medical Center Groningen, PO Box 196 (HPC FA21), 9700 AD Groningen, The Netherlands; 3 Fraunhofer Institute for Integrated Circuits IIS, Erlangen, Germany; 4 Cognitive Systems Group, Faculty Information Systems and Applied Computer Science, Otto-Friedrich University Bamberg, Bamberg, Germany

**Keywords:** Facial expression, Automatic pain detection, Pain diagnostics, Dementia

## Abstract

**Background:**

Given the unreliable self-report in patients with dementia, pain assessment should also rely on the observation of pain behaviors, such as facial expressions. Ideal observers should be well trained and should observe the patient continuously in order to pick up any pain-indicative behavior; which are requisitions beyond realistic possibilities of pain care. Therefore, the need for video-based pain detection systems has been repeatedly voiced. Such systems would allow for constant monitoring of pain behaviors and thereby allow for a timely adjustment of pain management in these fragile patients, who are often undertreated for pain.

**Methods:**

In this road map paper we describe an interdisciplinary approach to develop such a video-based pain detection system. The development starts with the selection of appropriate video material of people in pain as well as the development of technical methods to capture their faces. Furthermore, single facial motions are automatically extracted according to an international coding system. Computer algorithms are trained to detect the combination and timing of those motions, which are pain-indicative.

**Results/conclusion:**

We hope to encourage colleagues to join forces and to inform end-users about an imminent solution of a pressing pain-care problem. For the near future, implementation of such systems can be foreseen to monitor immobile patients in intensive and postoperative care situations.

## Background

### The need to identify pain in patients with dementia

There are an estimated 35.6 million people with dementia worldwide and this figure continues to rise. Treatment and care often fall below basic standards due to the unique and complex challenges presented by dementia. Identification and assessment of pain in people with dementia present a particular challenge [1]. It is thought that up to 80% of people with dementia living in care homes regularly experience pain from different causes [2]. The exact prevalence of pain is still difficult to determine because there are biasing factors like the beliefs of the caregivers how much pain is present in patients with dementia [3, 4] and the tendency of under-reporting pain in the group of patients with verbal communication problems [5]. Against this background, it is not surprising that epidemiological research has reported that the use of pain medication is often inappropriate in this patient group [6]. This is particularly prominent in care home and hospital settings where people are likely to have more severe cognitive impairment and are reliant on administration of analgesics by health professionals. A large number of studies have emphasized the challenge of assessing pain in people with dementia in these settings, and it is likely that this is the primary contributing factor to under-treatment of pain in these individuals [6].

Accordingly, thorough assessment of pain is essential to ensure effective treatment and ongoing care. In most patient groups the most effective method of identifying pain is through self-report. However, at moderate or severe stages of dementia, people with dementia often lack insight into their condition. In addition, a key symptom of dementia is the loss of ability to communicate, particularly in the later stages of the condition. These factors combined mean that people with dementia might not have the ability to give an accurate report of their pain [7]. As a result the majority of the usual pain assessment tools are partially inappropriate for use in dementia. Thus, a key element of any comprehensive assessment tool for dementia would be the observation of pain-related behaviors as necessary substitute for verbal report of pain, especially in moderate and severe dementia [8].

### The human observer of pain as substitute of the self-report of pain: promises and challenges

As mentioned above, research suggests that self-report seem to be valid in patients with mild degrees of dementia. However, self-report in persons with a score below 18 on the Mini Mental Status Examination (MMSE), indicative of moderate-to-strong dementia, may not be valid [9]. Thus, when dementia progresses and the person becomes less verbally communicative, observational pain tools become more important. Behavioral pain assessment tools typically focus on direct observation of pain-related behaviors, i.e. facial expression, body posture and movement, vocalization, etc., and also may include changes in behavior and functioning [8]. Research on pain in persons with dementia has largely been dominated by studies on the development of pain assessment tools, with a tremendous increase in attempts during the last two decades. The impressive number of development attempts shows the urgent need of appropriate tools and the insight that most tools are still far from perfect. But what are the promises and challenges so far?

#### Promises

Several studies suggest that the mere use of behavioral pain assessment tools in nursing homes and hospitals increases awareness of pain in health care professionals. In accord, they promote an improvement in the assessment and management of pain in dementia [7, 8]. Lukas et al. [10], for example, showed that the use of behavioral pain assessment tools improved recognition of the presence or absence of pain by over 25% above chance. The available behavioral pain assessment tools for patients with dementia have in common that they mostly focus on facial expression, body movements and vocalizations as sources of pain-related information. However, the single items used to assess pain-indicative behavior often vary immensely between scales. This means that there is agreement on the general sources of pain-relevant information in behavior but not on the specific behavioral indicators of pain [11].

Recent research suggests that amongst pain indicative behaviors (e.g. body movements, vocalization, etc.) the facial expression is one of the best suitable indicators of pain in cognitively unimpaired individuals as well as in older persons with dementia [12]. We and others could show that patients with dementia display the same type of facial movements when experiencing pain as cognitively unimpaired individuals do [13–15]. These facial movements include the contraction of the eyebrows, the contraction of the muscles surrounding the eyes, raising of the upper lip and opening of the mouth, to mention only a few of the most relevant [14]. Interestingly, observational pain assessment tools with items capturing these facial muscle movements demonstrated higher levels of sensitivity, reliability, and validity compared to scales that use more broad facial descriptors (e.g. looking tense) [12].

#### Challenges

Despite these promising developments in the last decades and despite the various observational pain assessment tools being available, pain assessment in patients with dementia is still challenging and often erroneous [7]. Why is that so? First of all, there is the time constraint. When healthcare professionals, mainly geriatric nurses, try to assess pain in patients with dementia their pain judgment is based on the time they interact with the patient, which can vary between a few seconds to a few minutes. However, a constant monitoring of the patient is not feasible. Furthermore, their monitoring is mainly centered on some activities of the daily living when the patients require more support and care. Thus, if the patient does not show any pain behavior during the limited time of interaction, pain that might be present during other times of the day remains undetected. Second and elaborating on the preceding argument, health care professionals, often try to observe the patients behavior while simultaneously performing the care (e.g. mobilizing or washing the patients). Thus, it is often not possible to observe the facial responses of the patients, whereas body movements and vocalization might be easier to detect. Third, pain indicative facial responses are often only subtle and fleeting. For example, the contraction of the muscles surrounding the eyes, which is the most frequent facial response to pain [16], is often quite difficult to detect and untrained human observers might not be able to make use of this facial movement to infer pain [17, 18]. Fourth, it is possible that psychotropic drug use, which is unfortunately very prevalent in people with dementia [19], or comorbidities like Parkinson Disease (PD), make it even more challenging to detect subtle facial expressions [20]. Fifth, observer biases might hinder correct pain assessment. Studies have shown, that professional observers consistently underestimate pain in others [21] and are not better in correctly identifying pain compared to individuals unfamiliar with the pain care of patients with dementia [22].

## Method/design

### A road map to find a better solution by use of a new interdisciplinary developed video-based system

One way to overcome some of the above mentioned challenges, and thus, improve pain assessment in patients with dementia, is to make use of an automatic video-based pain detection system as a complementary instrument supporting the human caregiver [7]. Such attempts are never meant to substitute the human observer or caregiver. Similarly as other monitors of vital functions (e.g. blood pressure, heart rate, respiration), a video-based pain monitor should unburden nurses and caregivers and thus, give them more time for the psychosocial and empathic side of human care.

Although automatic pain monitoring has also used modalities other than facial expressions, like physiological signals such as skin conductance, pupil dilatation and electrocardiogram (ECG) [23], the facial expression promises better discriminative validity and thus, better pain specificity compared to these other modalities, which mostly allow to indicate the level of arousal but not the specific type of distress. For that reason, we will focus on the detection of facial expression of pain.

The following sections of the manuscript will elaborate a kind of road map how to reach this goal to develop a video-based pain monitor focusing on the facial expression. One reason for writing a road map paper is to inform colleagues about our developmental strategies, technical solutions and adaptation to the needs of pain care in patients with dementia. By that, we would like to encourage them to join forces. This is to avoid unnecessarily scattered attempts of solution as was hampering the development of an internationally agreed on observational pain scale. Another reason for this road map paper is to inform the end-users, such as nurses and caregivers, about what might technically be possible in the future. They have to finally evaluate the feasibility of such automatic pain detection systems and should have visions of the potential optimization of pain care of patients with dementia.

In order to develop a system that is capable of identifying pain in patients with dementia, it is crucial to apply an interdisciplinary approach that comprises expertise in basic as well as applied research in the fields of pain, communication, dementia, facial expression, video image analyses, data analyses, machine learning as well as in clinical aspects of pain assessment in patients with dementia. In the following, we will describe our interdisciplinary approach and compare it to previous approaches.

The technical system for automatic detection of facial expressions envisaged in this article outputs Action Unit intensities based on the Facial Action Coding System. On the other side, the authors of this article from the psychology domain have in the past significantly contributed to the establishment of facial expression of pain data sets with cognitively healthy as well as impaired subjects. These data sets have been manually annotated with FACS Action Units and significant contributions to the understanding of facial expression of pain have been made, see e.g. [14]. In our joint preliminary work, based on this data, we already successfully qualitatively tested the feasibility of pain recognition in cognitively impaired elders. The complementary competencies, the availability of suitable data sources and the common language given by the Facial Action Coding System promote our belief that we can leverage automatic pain recognition in the cognitively impaired following our joint roadmap.

#### Step of choosing the right assessment criteria

In the last decade, several attempts have been made to develop automatic pain detection systems, with different aims of assessment. These were:

- Differentiation: pain versus no pain [24, 25].
- Differentiation: genuine versus faked pain [26, 27].
- Differentiation: pain versus other emotions [28, 29].
- Differentiation of different pain intensity: continuous-valued [30, 31] or discrete-valued [32–35].

In our case, the first and foremost assessment criteria of the system must be that it is able to differentiate between pain and other affective states, which are similar to pain and common in dementia (e.g. agitation, disgust). Indeed, in clinical practice health care professionals are mostly challenged with the decision whether a facial expression is indeed indicative of pain or of another affective state. A decision affirming the presence of pain activates an action scheme to help, cure and console [36]. Only in a next step, the graduating judgment of the pain intensity level becomes important. Thus, the first objective of an automatic pain detection system for patients with dementia should be to correctly answer the question: Is that person in pain or is the facial expression due to other forms of distress? The second objective will be to graduate the intensity of pain.

#### Step of selecting the appropriate training material

In line with the choice of the right assessment criteria, appropriate training material must be available, requiring a research group, which samples sufficient amounts of video data relating to the facial expression of pain and other sources of distress (e.g. anger, disgust, fear). The psychologists of our group were not only able to provide this data material but also to analyze it by use of the Facial Action Coding System (FACS) [37]; the code (Action Units) of which allows later for quantitative comparison between human coder and video-system. It should be noted that FACS coding requires a trained expert and is very time consuming. Thus, this approach is not suitable for application in nursing.

Especially, the differentiation of pain from general distress is challenging. As has been shown, observational tools measuring pain and those measuring distress overlap greatly in the content of their items [38]. Thus, videos of facial expression recorded in a distressful situation not containing pain should also be used for the training of the video-system. This has hardly yet been done, given that most automatic detection systems were customized by using only individuals experiencing either pain or no pain [24, 25] or only different intensities of pain [30–35]. However, only by testing the sensitivity and specificity of separating facial expressions of pain from other distress states, can the diagnostic performance of such an automatic pain detection system be sufficiently described. Moreover, it is important not to use actor portrayals as has often been done, because posed expressions differ substantially from spontaneous expressions as to be found in clinical situations. Spontaneous facial expressions differ from posed expressions in types of muscles being moved and in the dynamics of the movement. Thus, advances in the field of automatic pain detection systems must use spontaneous facial expressions [39]. Given that is ethically impossible to induce different distress states in patients with dementia, one should start with training the automatic detection system with video recordings of cognitively healthy individuals of different age groups. However, given that the automatic pain detection systems are developed with the aim to detect pain in cognitively impaired elders, these systems must also be tested in this target group. This is crucial because age changes in the skin structure (e.g. permanent wrinkles) and dementia-related comorbidities (e.g. Parkinson disease, stroke) can significantly impact the performance of these systems. Thus, we have started to use our video recordings of patients with dementia [14] to test the feasibility of our automatic pain detection system to detect pain in cognitively impaired elders. To the best of our knowledge, no previous work on automatically discerning pain from facial expressions was conducted in cognitively impaired elderly.

#### Technical steps

**Fig. 1:**
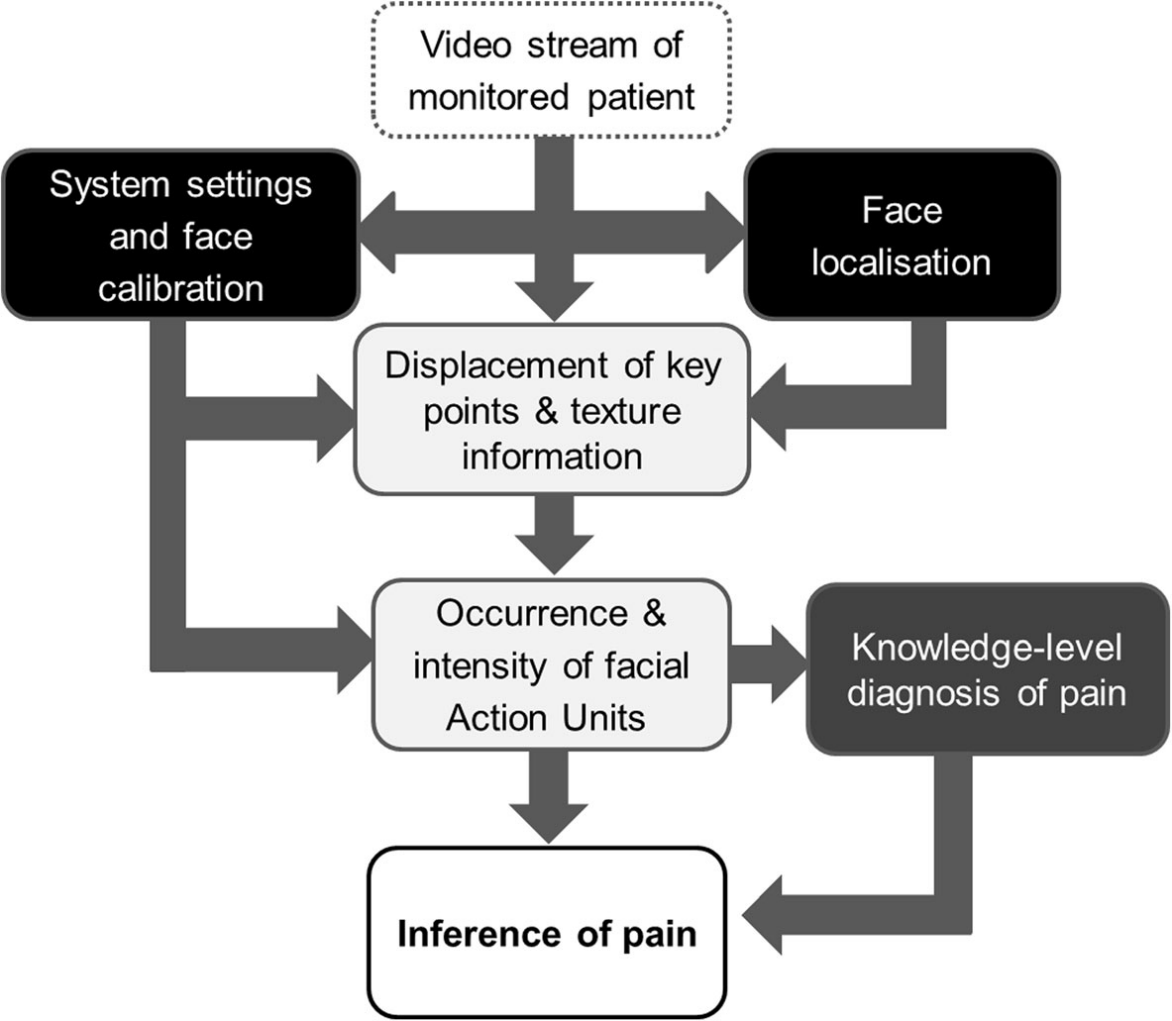
Technical steps necessary to develop an automatic system that is capable of identifying pain from facial expressions in patients with dementia

In the last 20 years, major advances have been achieved in computer vision research for automatic recognition of facial expressions, with necessary and good progress in different areas of this field. Three of these areas have been targets of further improvement for developing an automatic pain detection system by the engineers of our interdisciplinary group. These are: (i) capturing the face, (ii) analyzing facial motions and (iii) applying knowledge-level diagnosis of pain. Figure 1 tries to give a systematic overview over these three areas, with the different colored boxes (black, light grey and dark grey) representing the three areas.

- Step of robust face capturing (black colored boxes of Fig. 1)
  We use the “Sophisticated High Speed Object Recognition Engine” (SHORE, [40, 41]) developed by Fraunhofer IIS for detection of faces. Frontal face detection rate of the SHORE system is 91.5% with 10 false positives when tested on the public CMU + MIT data set (http://vasc.ri.cmu.edu/idb/html/face/frontal_images/index.html). This data set contains 507 annotated faces in 130 grayscale images. The face detection is based on local census and structure features. For classification, a classifier cascade is used (for more details see [40]) and together with a coarse-to-fine grid search this leads to an efficient real-time face detector. SHORE is also able to detect four basic emotions (anger, happiness, surprise, sadness) as well as valence (hedonic tone of the feeling (positive vs. negative)) [42]. Within our framework the SHORE system is used to locate the person’s face as well as the position of eyes, nose and mouth corners in each image of the video stream. The face is then normalized with respect to rotation and scaling. Thus, the normalized image always has the same resolution and pose. In this way, at least some of the variations in the appearance of the face that are caused by head rotations and movements of the person in front of the video capture device are mitigated, making this approach robust enough for capturing faces of bedridden patients. If more than one face is present, then the face detector selects the most prominent face on the basis of the face size in the image. If no face is detected, then the frame is not processed further.
- Step of analyzing single facial motions (light grey colored boxes of Fig. 1)
  Automatic detection of pain and pain levels from facial expressions is generally performed as a single or two-level detection process. In the former case, image sequences are processed directly when sequences can be supposed to be indicative of pain to extract characteristic features (e.g. [24, 31, 35]). In the latter case, image sequences are first processed for detecting single facial motions and coding them in terms of FACS (namely as AUs). Then (in a second step), the detected AUs and their intensities are processed to determine the likelihood of the presence and intensity of pain according to some thumb rules based on the available literature [26, 34]).
  Color or grayscale image sequences are commonly used as input to pain detection systems [24, 26, 30, 35]. More recently, depth and thermal images are also being used in combination with color images [43]. Numerical features describing the geometric shape or textural appearance of the face are extracted from each image in a sequence. The shape and appearance features are often used in combination [30, 31]. To incorporate expression dynamics, features are extracted over multiple images within a certain time interval [35]. In the two-level pain detection process, temporal features are also extracted from AU intensities [26]. The extracted features are processed using various machine learning methods in order to detect pain.
  In contrast to other approaches, our AU detection implements a temporal state model that connects each frame to the next [44]. This leads to a logical connection between successive frames, and with this property the system is able to mitigate noise and effectuate a temporal smoothing of the output. It is worth noting that the visco-elastic properties of facial muscles are taken into account in our state model by an individual mass-spring-damper model per AU. For the detection of the intensity of AUs in each frame, two sources of information are used.^1^ The geometric displacement of key points of the face (e.g. mouth corners) and texture information (e.g. wrinkles) are fused within the framework to make a final decision on the intensities of a selected set of AUs. During this process, an internal model of the facial morphology of the person is also taken into account. This model of the person’s “neutral” face is determined over time and helps to calibrate the system to the person’s face at runtime automatically [44]. This online calibration is necessary because it is often not possible to acquire a neutral face on demand. So in comparison to other approaches, we do not rely on an explicit calibration phase using a static mean face as a neutral face, since we think that this is not precise enough and will cause problems in distinguishing subtle expression related changes in the face from calibration errors.
- Step of applying knowledge-level diagnosis of pain (dark grey boxes of Fig. 1)
  Based on the identification of the temporal sequence of AUs and their intensities, a knowledge-level model can be built for diagnosis (see Fig. 1) – that is, the decision whether a patient experiences pain during the present video segment. Input in such a model is a pattern of AUs and output is the diagnosis. The diagnosis is performed by means of the application of symbolic rules, which represent patterns of AUs which are indicative for pain. Because the rules are represented symbolically, the diagnostic decision can be explained to a human observer.
  Diagnosis can be based either on prototypical, group-specific or individual patterns of AUs. Although a distinctive pain-indicative set of prototypical facial muscle movements has been identified that is displayed universally during pain [45], there are also substantial variations between individuals. We recently demonstrated that facial expressions of pain are best described as four distinct facial activity patterns of pain, shown reliably by certain groups of individuals, rather than as one single prototypical set of movements [46]. The most stable and most frequent patterns were ‘narrowed eyes’ combined with either (I) ‘wrinkled nose’ and ‘furrowed brows’; (II) ‘furrowed brows’ or (III) ‘opened mouth’ (the fourth pattern was not stable enough for further consideration). We could show that the most prominent facial movement which is part of each facial activity pattern, namely the ‘narrowed eyes’ encodes the sensory dimension of pain, whereas ‘wrinkled nose’ and ‘furrowed brows’ encode the affective dimension of pain [16]. Given these findings, the knowledge-based model will consider these three distinct facial activity patterns as well as consider whether a facial response might be indicative of pain intensity or pain affect in the diagnosis process. We hope that incorporating this knowledge in the automatic diagnosis process will improve sensitivity and specificity. By analogy, human observers benefit in their recognition aperformance from becoming aware of the presence of different facial activity patterns indicative of pain [47].
  The knowledge-based model is constructed either by classifier learning or by unsupervised learning (e.g. [48]). In the first case, a training set needs to include AU sequences observed for pain episodes as well as for non-pain episodes (e.g., disgust), the classifier is trained such that the rules have high sensitivity as well as high specificity for pain [49, 50]. In the second case, only pain episodes characteristic patterns are identified [50]. To exploit as much information as possible from the observed AU sequences, a rich representation language which also allows including domain specific knowledge as a background theory is helpful. Therefore, we currently investigate the application of inductive logic programming (ILP [51]) to learn diagnostic rules. In this framework, it is possible to learn rules which either only include information about the presence and possibly the intensity of specific AUs or rules which take into account information about sequences and simultaneous occurrence of AUs. A first empirical investigation indicates that human observers take sequential information into account [52]. At later stages of the process, we intend to use knowledge level diagnosis of pain that can be extended to sub-group classification learning. For example, knowing that facial expressiveness to pain is increased in patients with Alzheimer’s disease (AD) [14] and reduced in patients who are suffering from Parkinson Disease (PD) [20], we will at later stages apply sub-group classification learning (separately for patients with AD and PD), to possibly account for these pathological alterations.

### Testing the feasibility of our system in care settings of elders with dementia

There are mainly two stages of development. Stage 1 includes sampling of videos during care in situations of daily living with spontaneous or guided movements, which likely produce pain. This stage may be accompanied by video recordings during assessment of pressure pain sensitivity (e.g. simple palpation). The recording will be done by a camera man, who has to ensure ideal recording conditions. Since this approach is very time and staff consuming, in stage 2, a living lab with an appropriate multi-camera system will be used, in which senior home residents spend part of the day. Such a system provides a broader data base because - besides pain episodes provoked by a caregiver through guided movement -, spontaneous pain can no longer be missed due to continuous recording. Later on, night recording by ultraviolet cameras might be envisioned. The videos will be examined first offline. However, should move more and more towards the time constraints of online analyses.

## Discussion/conclusions

In order to provide adequate pain treatment in patients who are not able to self-report pain, observation of pain behavior, such as pain-indicative facial expressions, is crucial to detect pain in these patients. Given that a constant monitoring or observation of the patient by health care professionals is not possible, automatic pain detection systems are necessary. In this road map paper we describe an interdisciplinary approach to develop such a video-based pain detection system that focuses on one of the most prominent pain behaviors, namely the facial expression. The development starts with the selection of appropriate video material of people in pain as well as the development of technical methods to capture their faces. Furthermore, single facial motions are automatically extracted according to an international coding system. Computer algorithms are trained to detect the combination and timing of those motions, which are pain-indicative.

With this road-map paper, we hope to encourage colleagues to join forces and to inform end-users about an imminent solution of a pressing pain-care problem with the result of international and interdisciplinary collaborations. Finding relevant partners to form successful collaborations should not be very difficult because the number of key players in that field is not high. Indeed, appropriate consortiums like ours with expertise in the domains of pain, dementia, video-based systems, machine learning and computer-assisted diagnostics are rare. We hope that with the present road-map paper we will enforce dissemination of the topic to relevant key players as well as later end-users. However, we will also engage in other forms of dissemination. As next, incentives for the actual joining of forces have to be found. Nowadays, there are several instruments/calls available to fund large-scale projects at least at the European level, which require joining forces of the best key players for being successful. Therefore, nowadays staying aside and trying to compete may run into a greater risk than joining forces.

### Examples of future use and implementation

One of the first applications of our envisaged video system can be expected in patients with dementia who are immobile and are lying in bed. Given the technical solutions available at the moment, the reliable capture of the face will be possible only when the range of motions of the face is limited. Patients lying in bed can be expected to present enough facial aspects in frontal and lateral views to allow our video systems detecting the relevant facial expressions of pain. Is there need for such limited applications?

Unfortunately, the end-of-life constitutes a phase when pain often tremendously reduces the quality of life and hereby prevents dignified dying [53], because under-treated pain causes unnecessary suffering whereas over-treated pain (too much analgesics) cause unnecessary sedation. These problems are augmented in patients with dementia because they cannot report about pain and thus, make it more challenging to titrate the best possible dosage of analgesics [53]. In this palliative phase, there is definitely urgent need for a support that helps caregivers to decide about the appropriate dosage of medication for patients.

Another example of adequate application of our video system is the postoperative phase of pain monitoring. Dementia does not protect from physical causes of surgery (e.g. hip fractures). The sufficient management of acute postoperative pain is nowadays possible in many cases to avoid undue suffering and development of chronic postoperative pain. However, this adequate pain management requires the active assistance of the vigilant patient. The advantage of patient-controlled analgesia (PCA), when the patient controls behaviorally the dosage of analgesics, has been well documented [54]. However, PCA requires the patients to be active and vigilant (to decide which dosage of analgesics is sufficient) and thus, is mainly possible in cognitively unimpaired patients. Given the possibilities of our video system, this limitation might no longer apply, because this system might also help to titrate the necessary dosage. The urgent need for such systems might be further demonstrated by the fact that elderly and especially cognitively impaired patients are more likely to become delirious in the postoperative phase due to surgery or the aftereffects of anesthesia, preventing their active contribution to dosage finding. Also in these cases, our video-system may be excellent support of decision making as regards the appropriate pain management because the immobile and supine delirious patient provides best prerequisite for its application.

The scope of application will be widened as soon as more active systems including maneuverable swivel arms become available which allow targeting the face of more mobile patients over a wide range of motions.

## Abbreviations

AD: Alzheimer’s disease
AUs: Action units
ECG: Electrocardiogram
FACS: Facial action coding system
MMSE: Mini mental status examination
PCA: Patient-controlled analgesia
PD: Parkinson disease
SHORE: Sophisticated high speed object recognition engine

## Footnotes

1 Note that the number of information sources is not limited to these two since we implemented a multi sensor framework that allows us to easily add new types of information sources.

## References

[1] Husebo BS, Kunz M, Achterberg WP, Lobbezoo F, Kappesser J, Tudose C, Lautenbacher S (2012). Pain assessment and treatment challenges in patients with dementia. Zeitschrift für Neuropsychologie.

[2] Miro J, Paredes S, Rull M, Queral R, Miralles R, Nieto R, Huguet A, Huguet J (2007). Pain in older adults: a prevalence study in the mediterranean region of Catalonia. Eur J Pain.

[3] Kappesser J, Williams ACDC (2010). Pain estimation: asking the right questions. Pain.

[4] Engle VF, Graney MJ, Chan A (2001). Accuracy and bias of licensed practical nurse and nursing assistant ratings of nursing home residents’ pain. J Gerontol A Biol Sci Med Sci.

[5] Husebo BS, Kunz M, Achterberg WP, Lobbezoo F, Kappesser J, Tudose C, Lautenbacher S (2012). Pain assessment and treatment challenges in patients with dementia. Zeitschrift für Neuropsychologie.

[6] Achterberg WP, Pieper MJC, van Dalen-Kok AH, de Waal MWM, Husebo BS, Lautenbacher S, Kunz M, Scherder EJA, Corbett A (2013). Pain management in patients with dementia. Clin Interv Aging.

[7] Hadjistavropoulos T, Herr K, Prkachin KM, Craig KD, Gibson SJ, Lukas A, Smith JH (2014). Pain assessment in elderly adults with dementia. Lancet Neurol.

[8] Zwakhalen SM, Hamers JP, Abu-Saad HH, Berger MP (2006). Pain in elderly people with severe dementia: a systematic review of behavioural pain assessment tools. BMC Geriatrics.

[9] Chibnall J, Tait R (2001). Pain assessment in cognitively impaired and unimpaired older adults: a comparison of four scales. Pain.

[10] Lukas A, Barber JB, Johnson P, Gibson SJ (2013). Observer‐rated pain assessment instruments improve both the detection of pain and the evaluation of pain intensity in people with dementia. Eur J Pain.

[11] Corbett A, Achterberg W, Husebo B, Lobbezoo F, de Vet H, Kunz M, Strand L, Constantinou M, Tudose C, Kappesser J, de Waal M, Lautenbacher S (2014). An international road map to improve pain assessment in people with impaired cognition: the development of the Pain Assessment in Impaired Cognition (PAIC) meta-tool. BMC Neurology.

[12] Sheu E, Versloot J, Nader R, Kerr D, Craig KD (2011). Pain in the elderly: validity of facial expression components of observational measures. Clin J Pain.

[13] Kunz M, Mylius V, Scharmann S, Schepelman K, Lautenbacher S (2009). Influence of dementia on multiple components of pain. Eur J Pain.

[14] Kunz M, Scharmann S, Hemmeter U, Schepelman K, Lautenbacher S (2007). The facial expression of pain in patients with dementia. Pain.

[15] P. A. Beach, J. T. Huck, M. M. Miranda, K. T. Foley, and A. C. Bozoki. Effects of Alzheimer’s Disease on the Facial Expression of Pain. The Clinical Journal of Pain, in press. 10.1097/AJP.0000000000000302 26379075

[16] Kunz M, Lautenbacher S, Leblanc N, Rainville P (2012). Are both the sensory and the affective dimensions of pain encoded in the face?. Pain.

[17] Kunz M (2015). Do observers use the same facial movements that encode pain when inferring pain in others?. Eur J Pain.

[18] Roy C, Blais C, Fiset D, Rainville P, Gosselin F (2015). Efficient information for recognizing pain in facial expressions. Eur J Pain.

[19] Nijk RM, Zuidema SU, Koopmans RT (2009). Prevalence and correlates of psychotropic drug use in Dutch nursing-home patients with dementia. Int Psychogeriatr.

[20] Priebe A, Kunz M, Morcinek C, Rieckmann P, Lautenbacher S (2015). Does Parkinson’s disease lead to alterations in the facial expression of pain?. J Neurol Sci.

[21] Prkachin KM, Solomon PE, Ross J (2007). Underestimation of pain by health-care providers: towards a model of the process of inferring pain in others. CJNR (Canadian Journal of Nursing Research).

[22] Lautenbacher S, Niewelt BG, Kunz M (2013). Decoding pain from the facial display of patients with dementia: a comparison of professional and nonprofessional observers. Pain Med.

[23] A. Temitayo. M.S. Olugbade, H. Aung, N. Bianchi-Berthouze, N. Marquardt, and A. C. Williams. Bimodal detection of painful reaching for chronic pain rehabilitation systems. In Proceedings of the 16th International Conference on Multimodal Interaction, ICMI ‘14, pp. 455–458, New York, NY, USA, 2014.

[24] P. Lucey, J.F. Cohn, I. Matthews, S. Lucey, S. Sridharan, J. Howlett, and K.M. Prkachin. Automatically Detecting Pain in Video Through Facial Action Units. Systems. *Man and Cybernetics*, Part B: Cybernetics, IEEE Transactions on. 2011; vol. 41, no. 3, pp. 664–674 10.1109/TSMCB.2010.2082525 PMC6942457 21097382

[25] Zhang W, Xia L (2011). Pain expression recognition based on SLPP and MKSVM. Int J Eng Manag Econ (IJEM).

[26] Bartlett MS, Littlewort GC, Frank MG, Lee K (2014). Automatic decoding of facial movements reveals deceptive pain expressions. Curr Biol.

[27] Littlewort GC, Bartlett MS, Lee K (2009). Automatic coding of facial expressions displayed during posed and genuine pain. Image Vis Comput.

[28] Niese R, Al-Hamadi A, Panning A, Brammen D, Ebmeyer U, Michaelis B (2009). Towards pain recognition in post-operative phases using 3d-based features from video and support vector machines. JDCTA.

[29] Hammal Z, Kunz M (2012). Pain monitoring: a dynamic and context-sensitive system. Pattern Recognit.

[30] S. Kaltwang,, O. Rudovic, and M.Pantic. Continuous pain intensity estimation from facial expressions. In Advances in Visual Computing. Springer Berlin Heidelberg, pp. 368–377, 2012.

[31] P. Werner, A. Al-Hamadi, and R. Niese. Pain recognition and intensity rating based on comparative learning. In Image Processing (ICIP), 19th IEEE International Conference, pp. 2313–2316, 2012.

[32] Z. Hammal, and J.F. Cohn. Automatic Detection of Pain Intensity. In Proceedings of the 14th ACM International Conference on Multimodal Interaction, ICMI ‘12, New York, NY, USA, pp. 47–52, 2012. 10.1145/2388676.2388688 PMC7385931 32724903

[33] O. Rudovic, V. Pavlovic, and M. Pantic. Automatic pain intensity estimation with heteroscedastic conditional ordinal random fields. In Advances in Visual Computing. Springer Berlin Heidelberg, pp. 234–243, 2013.

[34] A. Ghasemi, X. Wei, P. Lucey, S. Sridharan, and C. Fookes. Social signal processing for pain monitoring using a hidden conditional random field. In Statistical Signal Processing (SSP), 2014 IEEE Workshop, pp. 61–64. IEEE, 2014.

[35] Irani R, Nasrollahi K, Simon MO, Corneanu CA, Escalera S, Bahnsen C, Lundtoft D, Moeslund TB, Pedersen T, Klitgaa ML, Petrini L (2015). Spatiotemporal analysis of RGB-DT facial images for multimodal pain level recognition.

[36] Herr K, Bjoro K, Decker S (2006). Tools for assessment of pain in nonverbal older adults with dementia: a state-of-the-science review. J Pain Symptom Manage.

[37] Ekman P, Friesen WV (1978). The facial action coding system.

[38] van der Steen JT, Sampson EL, Van den Block L, Lord K, Vankova H, Pautex S, Vandervoort A, Radbruch L, Shvartzman P, Sacchi V, de Vet HCW, Van den Noortgate NJA (2015). Tools to assess pain or lack of comfort in dementia: a content analysis. J Pain Symptom Manag.

[39] Bartlett MS, Littlewort GC, Frank MG, Lainscsek C, Fasel IR, Movellan JR (2006). Automatic recognition of facial actions in spontaneous expressions. J Multimed.

[40] Küblbeck C, Ernst A (2006). Face detection and tracking in video sequences using the modified census transformation. Image Vis Comput.

[41] T. Ruf, A. Ernst, C. Küblbeck. Face Detection with the Sophisticated High-speed Object Recognition Engine (SHORE). In Microelectronic Systems—Circuits, Systems and Applications, Springer, pp. 243–252, 2012

[42] Garbas J-U, Ruf T, Unfried M, Dieckmann A (2013). Towards robust real-time valence recognition from facial expressions for market research applications.

[43] M. Valstar. Automatic behaviour understanding in medicine. In Proceedings of the 2014 Workshop on Roadmapping the Future of Multimodal Interaction Research including Business Opportunities and Challenges, pp. 57–60, 2014.

[44] Hassan T, Seuss D, Wollenberg J, Garbas J, Schmid U. A Practical Approach to Fuse Shape and Appearance Information in a Gaussian Facial Action Estimation Framework. In Gal A. Kaminka (Ed.), Proceedings of European Conference on Artificial Intelligence (ECAI 2016), vol. 285, pp. 1812–1817, 2016, The Hague, The Netherlands.

[45] Prkachin KM (1992). The consistency of facial expressions of pain: a comparison across modalities. Pain.

[46] Kunz M, Lautenbacher S (2004). The faces of pain: a cluster analysis of individual differences in facial activity patterns of pain. Eur J Pain.

[47] M. Kunz, and S. Lautenbacher, S. Improving recognition of pain by calling attention to its various faces. European Journal of Pain, in press 10.1002/ejp.666 25736626

[48] T. Mitchell. Machine Learning. McGraw Hill, 1997.

[49] M. Siebers, M. Kunz, S. Lautenbacher, and U. Schmid. Classifying Facial Pain Expressions: Individual Classifiers vs. Global Classers. In Dirk Reichardt (Ed.), Proceedings of the 4th Workshop on Emotion and Computing—Current Research and Future Impact, 2009.

[50] Siebers M, Schmid U, Seuß D, Kunz M, Lautenbacher S (2016). Characterizing facial expressions by grammars of action unit sequences - a first investigation using ABL. Inform Sci.

[51] Muggleton S, De Raedt L (1994). Inductive logic programming: theory and methods. J Log Program.

[52] M. Siebers, T. Engelbrecht, and U. Schmid. On the Relevance of Sequence Information for Decoding Facial Expressions of Pain and Disgust {An Avatar Study. In: D. Reichardt (Hrsg.): Proceedings 7th Workshop Emotion & Computing. Current Research and Future Impact, pp. 3–9, 2013.

[53] Sampson EL, Ritchie CW, Lai R, Raven PW, Blanchard MR (2005). A systematic review of the scientific evidence for the efficacy of a palliative care approach in advanced dementia. Int Psychogeriatr.

[54] Wu CL, Cohen SR, Richman JM, Rowlingson AJ, Courpas GE, Cheung K, Lin EE, Liu SS (2015). Efficacy of postoperative patient-controlled and continuous infusion epidural analgesia versus intravenous patient-controlled analgesia with opioids: a meta-analysis. Anesthesiology.

